# Hormonal status and reproductive life-course states as modulators of women’s physiological responses to acute and chronic hypoxia: insights, challenges, and perspectives

**DOI:** 10.1186/s13293-026-00954-1

**Published:** 2026-07-18

**Authors:** Ayoub Boulares, Olivier Dupuy, Nicola Luigi Bragazzi, Carina Enéa, Nathalie Delpech, Philippe Connes, Paul Robach, Samuel Vergès, Aurélien Pichon

**Affiliations:** 1 Laboratory Mobility, Aging & Exercise-ER20296 (MOVE), Faculty of Sport Sciences-STAPS, University of Poitiers, Poitiers, France; 2 School of Kinesiology and Physical Activity Sciences (EKSAP), Faculty of Medicine, University of Montreal, Montreal, H3T 1J4 QC Canada; 3 Department of Mathematics and Statistics, Laboratory for Industrial and Applied Mathematics (LIAM), York University, Toronto, ON Canada; 4 Health Anthropology Biosphere and Healing Systems, United Nations Educational, Scientific and Cultural Organization (UNESCO) Chair, University of Genoa, Genoa, Italy; 5 Department of Clinical Pharmacy, Saarland University, Saarbrücken, 66123 Germany; 6 Laboratoire Interuniversitaire de Biologie de la Motricité (LIBM) EA7424, Team ‘Vascular Biology and Red Blood Cell’, Université Claude Bernard Lyon 1, Université de Lyon, Lyon, France; 7 Laboratoire D’excellence du Globule Rouge (Labex GR-Ex), PRES Sorbonne, Paris, France; 8 National School for Mountain Sports, Site of the National School for Skiing and Mountaineering (ENSA), Chamonix, France; 9 Univ. Grenoble Alpes, CHU Grenoble Alpes, Inserm, Grenoble, HP2, 38000 France

**Keywords:** Women, Hypoxia, High altitude, Reproductive health, Hormonal status, Hormonal contraception, Menstrual cycle, Menopause, Hormone replacement therapy, Gender-affirming hormone therapy

## Abstract

Hypoxia research has significantly advanced our understanding of how the human body responds to low-oxygen environments, yet women are still frequently studied without adequate consideration of hormonal status or reproductive life-course stage. This review examines the complex interactions between hormonal status across the female life course and the specific physiological responses of women to acute and chronic hypoxia, focusing on the menstrual cycle, hormonal contraception, pregnancy, menopausal status, hormone replacement therapy, gender-affirming hormone therapy, and reproductive health in high-altitude environments. Estrogen and progesterone significantly modulate ventilatory, cardiovascular, hematological, vascular, and muscular/metabolic responses to hypoxia, with distinct effects across different menstrual cycle phases, contraceptive regimens, and life-course states. Menopause introduces additional complexities, as declining hormone levels alter the body’s ability to acclimatize to low-oxygen conditions. Additionally, we examine how chronic and lifelong hypoxia impacts reproductive health, including fertility and pregnancy outcomes, in women living at high altitudes, highlighting both physiological adaptations and contextual factors. While current research has made progress, further studies are needed to better understand these sex-specific responses. We propose that future research should integrate stratified approaches, accounting for hormonal status (cycle phase, contraceptive use, pregnancy, HRT, GAHT) and reproductive status, to optimize health and performance recommendations for women exposed to hypoxic environments.

## Highlights

Hormonal status and reproductive life-course stage may modulate women’s physiological responses to hypoxia.

Hypoxic exposure models differ in dose, duration, mechanisms, and physiological interpretation.

Menstrual cycle phase, hormonal contraception, pregnancy, menopause, hormone replacement therapy, and gender-affirming hormone therapy represent distinct hormonal contexts.

Hormonal modulation may affect ventilatory, cardiovascular, vascular, hematological, metabolic, and placental responses.

Stratified study designs should report hormonal status, hypoxic dose, oxygen saturation response, exposure duration, and rest, exercise, or sleep context.

## Background

High-altitude environments, typically defined as regions situated at elevations above 1,500 m (4,900 feet), present unique physiological challenges primarily due to reduced atmospheric oxygen levels, a condition referred to as hypoxia [1]. However, hypoxia should not be considered a uniform physiological stimulus. It may occur across several exposure models, ranging from acute laboratory exposure and short-term altitude sojourns to repeated hypoxic training or therapeutic protocols, chronic high-altitude residence, and lifelong or multigenerational high-altitude exposure [2, 3]. These exposure models differ substantially in terms of hypoxic dose, severity, duration, and physiological interpretation. Acute hypoxia primarily elicits immediate ventilatory, cardiovascular, autonomic, and metabolic responses, whereas repeated or prolonged exposure may induce acclimatization processes involving ventilatory adaptation, hematological changes, vascular regulation, and oxygen-sensing pathways [4, 5]. Chronic or lifelong high-altitude exposure may further reflect developmental, genetic, environmental, and cultural influences [6–8]. Therefore, findings obtained in one hypoxic exposure model cannot be directly extrapolated to another without considering exposure duration, altitude level or inspired oxygen fraction, oxygen saturation, and cumulative hypoxic dose.

Extensive research has characterized general physiological responses to hypoxia, including increased ventilation, altered cardiovascular regulation, vascular adjustments, and enhanced erythropoiesis [9–11]. However, a critical gap persists in understanding how these responses are modulated in women according to hormonal status and reproductive life-course stage. Women are often studied as a homogeneous group, despite substantial endocrine variation across the menstrual cycle, hormonal contraceptive use, pregnancy, menopause, hormone replacement therapy (HRT), and other exogenous hormone exposures [12, 13]. This is an important limitation because ovarian and exogenous sex steroids influence several systems directly involved in hypoxic responses, including respiratory control, autonomic and cardiovascular regulation, vascular function, hematological responses, and metabolism [14–18].

Women’s reproductive physiology is intricately regulated by the Hypothalamic-Pituitary-Gonadal (HPG) axis, a sophisticated neuroendocrine system that governs the menstrual cycle, fertility, and menopausal transitions. Hormonal fluctuations across the menstrual cycle, driven by hormones such as gonadotropin-releasing hormone (GnRH), luteinizing hormone (LH), follicle-stimulating hormone (FSH), estrogen, and progesterone, play a pivotal role in regulating ovulation, menstrual bleeding, and overall physiological balance [19]. These hormones not only influence reproductive function but also have far-reaching effects on other systems, including the autonomic nervous system, respiratory control, and cardiovascular regulation, vascular function, hematological responses, and metabolism, all of which are directly involved in the body’s response to hypoxia [14, 15, 17]. Therefore, women’s responses to hypoxia should be interpreted in relation to their hormonal context rather than treated as uniform across all women. In naturally cycling women, this requires attention not only to broad menstrual cycle phases, but also to variations in the estrogen/progesterone ratio. In women using hormonal contraception or HRT, the type, dose, route, and timing of exogenous hormone exposure may also influence hypoxia-related outcomes.

Although recent studies have begun to examine women’s physiological responses to hypoxia and high altitude in relation to their hormonal status and menopausal state, the evidence remains limited and methodologically heterogeneous [20–22]. Many studies either do not differentiate between men and women or do not adequately characterize hormonal status within women. This lack of stratification may obscure sex- and hormone-specific differences in terms of physiological and biological responses to hypoxia. It may also partly explain inconsistent findings across studies, particularly when acute hypoxic responses, short-term acclimatization, repeated exposure, chronic exposure, and lifelong high-altitude adaptation are discussed together without sufficient distinction.

Reproductive life-course states also require careful interpretation in hypoxia research, as pregnancy, menopause, and long-term high-altitude residence represent distinct physiological contexts with different endocrine, cardiovascular, respiratory, and metabolic implications. Chronic hypobaric hypoxia during long-term or lifelong high-altitude exposure has been associated with a range of reproductive consequences, including delayed menarche, advanced menopause, and a shortened reproductive lifespan [21, 23, 24]. Women living at high altitudes also frequently experience menstrual irregularities, which may be linked to disruptions in the HPG axis induced by chronic hypoxia. Notably, fertility rates have been observed to increase when women relocate from high-altitude to low-altitude environments, suggesting a potentially reversible component to some hypoxia-related reproductive effects [24]. However, these observations should be interpreted within the specific context of chronic or lifelong altitude exposure and should not be directly extrapolated to acute or short-term hypoxic models. Menopause further illustrates the need to interpret hypoxic responses within a specific hormonal and life-course context. The menopausal transition, characterized by the progressive decline in ovarian hormone production, may alter ventilatory control, cardiovascular regulation, vascular function, and oxygen transport under hypoxic conditions [25]. However, menopause should not be reduced to the absence of ovarian hormones, as ageing, time since menopause, cardiometabolic status, and HRT may each influence hypoxia-related responses. Similarly, other hormonal contexts, including hormonal contraception and pregnancy, require specific consideration because they may modify physiological and reproductive outcomes during hypoxic exposure.

The interaction between hormonal status and hypoxia may also involve temporal regulation. High-altitude hypoxia can act as an environmental zeitgeber, serving as an external cue that influences biological clocks. However, rather than enhancing circadian regulation, hypoxia may disturb the synchronization between central and peripheral clocks, thereby altering circadian rhythms and hormonal regulation [26]. Such disruptions can interfere with the alignment between physiological adaptations to hypoxia and the body’s reproductive phases, potentially compromising reproductive function and overall homeostasis under low-oxygen conditions [27]. This interaction illustrates that hypoxic responses may depend not only on sex and hormonal status, but also on the temporal characteristics of exposure, including altitude level, inspired oxygen fraction, oxygen saturation, exposure duration, frequency, and the physiological condition under which exposure occurs, such as rest, sleep, or exercise.

This review aims to provide an integrated framework for understanding how hormonal status and reproductive life-course states modulate women’s physiological responses to hypoxia. Specifically, we consider menstrual cycle phase, hormonal contraception, pregnancy, menopausal status, HRT, and gender-affirming hormone therapy as hormonal contexts that may influence responses to low-oxygen environments. We also distinguish acute hypoxic responses from short-term acclimatization, repeated or intermittent exposure, chronic exposure, and lifelong high-altitude adaptation, because these conditions differ in terms of dose, duration, mechanisms, and physiological interpretation. By integrating hormonal status, hypoxic dose, exposure duration, and targeted physiological systems, this review aims to clarify current evidence and highlight the need for stratified research designs in women’s hypoxia research.

## Hypoxic exposure models and dose-response considerations

Hypoxia is not a uniform exposure, and the way it is induced or administered determines which physiological systems are primarily engaged and how hormonal status may modulate the response. This distinction is particularly important in women’s hypoxia research, because, as previously mentioned, menstrual cycle phase, hormonal contraception, pregnancy, menopause, HRT, and other exogenous hormone exposures may influence ventilatory, cardiovascular, vascular, hematological, metabolic, and reproductive responses differently depending on the hypoxic stimulus. For example, progesterone-related effects on ventilatory drive may be especially relevant during acute hypoxic exposure, whereas estrogen-related effects on erythropoiesis, endothelial regulation, and pulmonary vascular responses may become more apparent during repeated, chronic, or lifelong hypoxic exposure [28–30]. Therefore, findings obtained in one exposure model cannot be assumed to generalize to another.

Four broad exposure models can be distinguished (Table 1). Acute hypoxia, lasting from minutes to hours, captures immediate responses such as changes in ventilation, heart rate, autonomic regulation, vascular tone, and arterial oxygenation, without sufficient time to infer acclimatization. Short-term or repeated hypoxia, lasting from days to weeks, may engage early acclimatization processes, including ventilatory adaptation, initial vascular responses, and activation of oxygen-sensing pathways [31, 32]. Long-term repeated or intermittent hypoxia, extending over months to years, may induce more sustained changes in ventilatory control, endothelial function, oxidative-stress signaling, and metabolic regulation, with the magnitude and direction of these responses depending on exposure severity, frequency, and cumulative dose. Chronic or lifelong high-altitude exposure additionally involves hematological, pulmonary vascular, reproductive, developmental, and, particularly across generations, genetic, epigenetic, environmental, and sociocultural dimensions [31, 33, 34].

Beyond exposure duration, hypoxic dose is equally important for interpretation. Altitude level or inspired oxygen fraction, peripheral or arterial oxygen saturation, exposure frequency, and cumulative hypoxic burden all influence the magnitude and direction of physiological responses. In addition, normobaric and hypobaric hypoxia should not always be considered interchangeable, as they may differ in environmental characteristics and physiological effects. The context in which hypoxia occurs, including rest, sleep, or exercise, further modifies the physiological systems involved. These methodological dimensions should therefore be systematically reported and considered when interpreting how hormonal status modifies women’s responses to hypoxia.

**Table 1:** Hypoxic exposure models and interpretative considerations in women’s hypoxia research

Hypoxic exposure model	Typical duration	Examples	Main interpretation	Hormonal-status considerations	Essential reporting
Acute hypoxia	Minutes to hours	Normobaric hypoxia; hypobaric chamber exposure; rapid ascent; brief altitude exposure	Immediate responses to reduced oxygen availability; not sufficient to infer acclimatization or long-term adaptation	Cycle phase, contraceptive type, time-of-day hormones most relevant	FiO₂ or altitude equivalent, exposure duration, SpO₂ response, rest/exercise condition, posture, time of day, hormonal status
Short-term/repeated hypoxia	Days to weeks	Short altitude sojourn; repeated hypoxic sessions; intermittent hypoxic training; hypoxic exercise protocols	Early acclimatization or conditioning responses; cumulative effects depend on frequency, recovery, and total hypoxic dose	Cycle phase stability across sessions; contraceptive continuity	Number of sessions, duration per session, frequency, hypoxic/normoxic intervals, SpO₂ nadir, cumulative dose, exercise intensity, hormonal status across sessions
Long-term repeated / intermittent hypoxia	Months to years	Repeated altitude exposure; long-term hypoxic training; recurrent intermittent hypoxic exposure; sleep-related intermittent hypoxia when relevant	Sustained remodeling depending on exposure pattern, severity, and repetition; should be distinguished from both acute exposure and continuous high-altitude residence	Long-term contraceptive/HRT exposure; menopausal transition	Exposure pattern, frequency, cumulative dose, SpO₂ profile, sleep/exercise context, pathological vs. intervention-based exposure, hormonal status
Chronic/lifelong hypoxia	Years to generations	Long-term high-altitude residence; lifelong exposure; multigenerational high-altitude populations	Chronic acclimatization and/or developmental, genetic, epigenetic, and environmental adaptation; not directly comparable to acute laboratory hypoxia	Reproductive lifespan effects; menopausal age; pregnancy history	Altitude of residence, duration of residence, altitude of birth, ancestry, migration history, acclimatization status, hemoglobin/hematocrit, pregnancy/reproductive history, socioeconomic/healthcare context

FiO₂: Fraction of inspired oxygen, SpO₂: Peripheral oxygen saturation

## Hormonal status as a modifier of hypoxic responses

### Menstrual cycle and ovarian hormone fluctuations

Understanding the neuroendocrine mechanisms that mediate hormonal influences on hypoxic responses is crucial for elucidating sex-specific physiological adaptations. The HPG axis orchestrates the menstrual cycle through the coordinated secretion of GnRH from the hypothalamus, LH and FSH from the anterior pituitary gland, and the steroid hormones estrogen and progesterone from the ovaries [19]. These hormonal fluctuations, particularly during the follicular phase (Days 1–14) and the luteal phase (Days 15–28) of the menstrual cycle [13], exert profound impacts on the autonomic nervous system and the respiratory and cardiovascular systems [14, 15, 17], which are directly involved in the body’s response to hypoxia [35]. During the follicular phase, rising estrogen levels promote vasodilation and enhance blood flow, thereby increasing tissue oxygenation, and ovarian hormones have been shown to modulate ventilatory responses to hypoxia, in part by enhancing peripheral chemoreceptor sensitivity to low oxygen [16, 36]. This heightened blood flow ensures that oxygen delivery to vital organs is optimized, which is essential when oxygen availability is compromised. Around mid-cycle (typically ~ Day 14 in a 28-day cycle), rising estradiol levels trigger a positive feedback loop resulting in a surge of LH. This LH surge stimulates ovulation, causing the dominant follicle to release the oocyte, and initiates the transformation of the ruptured follicle into the corpus luteum, marking the onset of the luteal phase [13, 37].

In the luteal phase, elevated progesterone levels act as potent respiratory stimulants by increasing minute ventilation [38], which enhances the body’s capacity to take up oxygen more efficiently during hypoxic stress. Progesterone achieves this by upregulating Hypoxia-Inducible Factors (HIF-1α and HIF-2α), transcription factors that activate the expression of genes involved in angiogenesis, erythropoiesis, and metabolic adaptation, thereby facilitating cellular and systemic adaptations to low-oxygen environments [39]. Conversely, estrogen downregulates HIF-2α [40], consequently attenuating certain hypoxic responses to prevent excessive angiogenesis and metabolic shifts when oxygen demand is lower, serving as a balancing mechanism to maintain physiological homeostasis.

Moreover, progesterone enhances the sensitivity of both central and peripheral chemoreceptors, ensuring that ventilatory responses are appropriately scaled to the body’s needs during hypoxic conditions. In contrast, estrogen modulates chemoreceptor sensitivity in relation to reproductive and metabolic states, with evidence suggesting that during the luteal phase, characterized by elevated estrogen and progesterone levels, ventilatory responses to hypoxia are heightened, whereas during the follicular phase, when estrogen predominates before ovulation, ventilatory responses may be relatively lower [30]. Possible interactions between sex hormones and erythropoietin (EPO) have also been shown in hypoxic ventilatory response, suggesting an interplay between specific hypoxic physiological responses to hypoxia and menstrual phases [41, 42].

Beyond estrogen and progesterone, androgens are powerful modulators of erythropoiesis under hypoxic stress. Testosterone up-regulates renal EPO synthesis, establishes a higher EPO–hemoglobin set-point, and lowers hepcidin levels, thereby increasing systemic iron availability for erythropoiesis and accelerating red-cell mass expansion and oxygen-carrying capacity during sustained hypoxia [18, 43]. In women, circulating testosterone is normally low, but up to one woman in five in the USA presents “at-risk” hyperandrogenism, most commonly due to polycystic ovary syndrome (PCOS) [44]. Chronically elevated testosterone in these women is expected to augment erythropoiesis and potentially blunt the ventilatory response to hypoxia, a hypothesis still largely untested. An exaggerated rise in haematocrit might improve arterial O₂ content yet increase blood viscosity, alter carotid-body chemotransduction, and shift ventilatory acclimatisation curves. Prospective studies that stratify women by androgen status (e.g., PCOS, athletic hyperandrogenism, gender-affirming testosterone therapy) are therefore needed to determine whether high-testosterone phenotypes display a distinct hypoxia-response endotype. Beyond androgen-related mechanisms, ovarian steroids can also act directly on hematopoietic and erythroid progenitor programs, providing an additional mechanistic layer linking women’s hormonal milieu to hypoxia-related hematological adaptation.

Female sex hormones also directly modulate hematopoiesis and erythropoiesis through receptor-dependent mechanisms in hematopoietic and erythroid progenitor compartments. Estrogen signaling, primarily via estrogen receptors (ERα/ERβ), has been implicated in regulating hematopoietic stem/progenitor cell dynamics and erythroid output in a context-dependent manner. For instance, physiological increases in estrogen (e.g., pregnancy) have been associated with enhanced HSC cycling and expansion, with downstream effects on erythropoietic activity in peripheral hematopoietic sites [45]. Conversely, under chronic hypoxic stress, estrogen may act as a brake on excessive erythrocytosis by directly targeting key erythroid transcriptional programs. Using an in vitro erythroid differentiation platform derived from chronic mountain sickness (Monge’s disease), Azad et al. [28] showed that estradiol markedly reduced erythroid maturation (CD235a+ cells) in a stage- and dose-dependent manner, and mechanistically downregulated the erythroid transcription factor (GATA-1) at both mRNA and protein levels, together with canonical GATA-1 target genes involved in erythroid differentiation and survival (e.g., ALAS2, BCL-xL, EPOR), resulting in increased apoptosis of erythroid cells [28]. These effects were mediated predominantly through ERβ signaling and were accompanied by changes in hypoxia-related pathways, highlighting a mechanistic interface between sex-steroid signaling, erythroid transcriptional control, and hypoxia biology [28]. At the molecular level, earlier work supports the concept that estrogen receptors can interact with erythroid transcriptional machinery (including GATA-1) to modulate erythropoiesis in a ligand- and stage-dependent fashion [46]. Collectively, these data suggest that fluctuations in ovarian steroids across the menstrual cycle, and their decline after menopause, may contribute not only to ventilatory and cardiovascular variability, but also to inter-individual differences in hypoxia-related hematological phenotypes, particularly in settings of sustained or repeated hypoxic exposure [47].

In addition to their effects on erythropoiesis, ovarian hormones also modulate blood rheology and nitric oxide–dependent vascular regulation, with important cardiovascular implications under hypoxic conditions. Fluctuations in ovarian hormones across the menstrual cycle may also influence blood rheology and nitric oxide (NO)–dependent vascular regulation, with potential cardiovascular consequences under hypoxic conditions. Estrogen has been shown to modulate several determinants of blood viscosity, including plasma volume, hematocrit, and erythrocyte deformability, resulting in cyclical variations in whole-blood viscosity across the menstrual cycle [48]. Lower blood viscosity and improved red blood cell deformability have been reported during phases of elevated estrogen, which may facilitate microvascular perfusion and oxygen delivery, particularly when oxygen availability is reduced. In parallel, estrogen strongly stimulates endothelial NO synthase (eNOS) activity via both genomic and non-genomic mechanisms, thereby increasing NO bioavailability, promoting vasodilation, reducing vascular resistance, and enhancing shear–stress–mediated blood flow [49]. These effects are especially relevant in hypoxic environments, where NO plays a central role in matching oxygen delivery to metabolic demand and in limiting hypoxia-induced vasoconstriction [49]. Experimental and clinical evidence further suggests that variations in estrogen levels influence cerebrovascular and systemic vascular reactivity through NO-dependent pathways, with implications for blood pressure regulation and cardiovascular risk [50]. Collectively, hormone-driven changes in blood viscosity and NO signaling may contribute to inter-individual and phase-dependent differences in cardiovascular adaptation to hypoxia across the menstrual cycle, complementing ventilatory and hematological mechanisms.

**Fig. 1:**
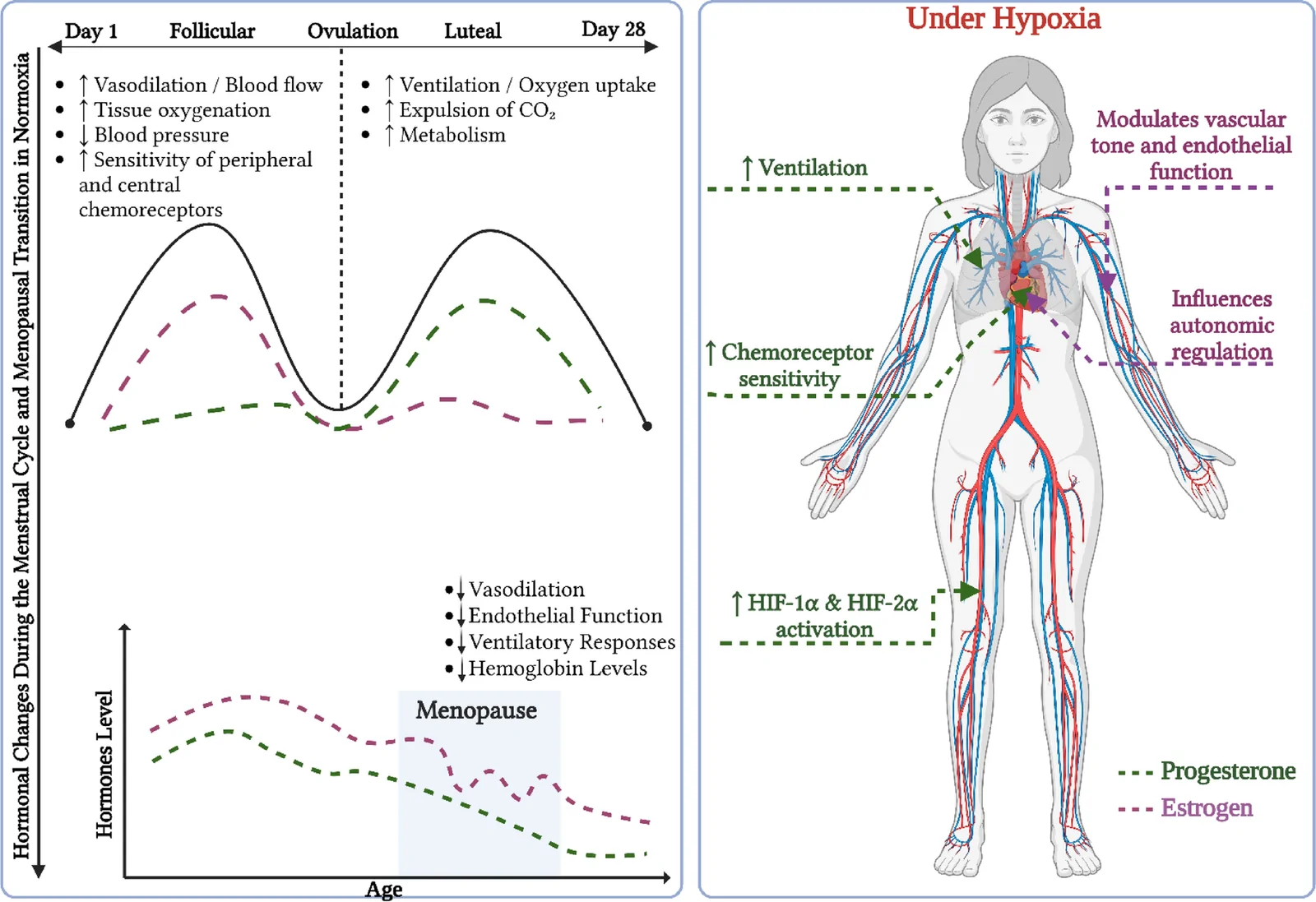
Hormonal fluctuations across the menstrual cycle and menopausal transition and their physiological implications under normoxia and hypoxia

Additionally, recognizing zeitgebers—external and internal cues such as hypoxia and the menstrual cycle—as synchronizing signals for internal biological clocks provides a comprehensive framework for interpreting the variability in hypoxic responses observed in women. Hypoxia, acting as an environmental zeitgeber, induces adaptive physiological responses necessary for survival in low-oxygen conditions, including increased ventilation, adjusted heart rate and cardiac output, and optimized blood flow distribution [51–53] (Fig. 1). Concurrently, the menstrual cycle, driven by the HPG axis, functions as an intrinsic zeitgeber that aligns hormonal fluctuations with circadian rhythms, thereby fine-tuning physiological adaptations to hypoxia based on reproductive phases. Importantly, the relationship between the menstrual cycle and hypoxia is bidirectional. While hormonal fluctuations modulate hypoxic responses, hypoxic exposure itself, particularly acute or chronic exposure to high-altitude environments, can disrupt ovarian circadian rhythms and reproductive hormone secretion. Recent findings by Ding et al. [54] demonstrate that acute hypoxia alters the expression of core clock genes in the ovary, leading to dysregulation of steroidogenesis and ovulation. This disruption can contribute to menstrual irregularities, such as oligomenorrhea or anovulation, especially in women exposed to prolonged or intense hypoxic conditions. Furthermore, menstrual disorders like menorrhagia, which have been reported more frequently at high altitudes, can exacerbate the risk of iron-deficiency anemia. In a hypoxic environment where oxygen-carrying capacity is already challenged, coexisting anemia may further impair physiological tolerance and ventilatory responses, underscoring the importance of comprehensive evaluation of women’s reproductive health in hypoxic research and clinical management.

This intricate interplay between the HPG axis and hypoxia-induced signaling pathways highlights the importance of considering both hormonal cycles and environmental cues in understanding and predicting women’s physiological adaptations to low-oxygen environments. Such an integrated approach not only elucidates the underlying mechanisms of sex-specific hypoxic responses but also highlights the necessity for personalized strategies in clinical and high-altitude settings to optimize health and performance outcomes for women living in hypoxic environments.

### Hormonal contraception

Hormonal contraceptive use is widespread among women of reproductive age, yet its influence on physiological responses to hypoxia remains markedly understudied. Unlike naturally occurring menstrual cycles, in which estradiol and progesterone fluctuate cyclically, hormonal contraceptives modify the endogenous HPG axis through exogenous steroid exposure and negative feedback on gonadotropin secretion [55]. Combined hormonal contraceptives, including combined oral contraceptive pills, transdermal patches, and vaginal rings, generally suppress ovulation through negative feedback on gonadotropin secretion and provide relatively stable exposure to synthetic estrogen and progestins [56]. Progestin-only methods, including progestin-only pills, implants, injectables, and hormonal intrauterine devices, differ substantially in systemic progestin exposure, degree of ovulation suppression, bleeding profile, and potential metabolic effects [57, 58]. Failure to account for contraceptive status may therefore contribute to inter-study heterogeneity and may partly explain inconsistent findings regarding menstrual cycle phase and hypoxic responses across cohorts. Accordingly, hormonal contraception should not be treated as a single homogeneous category in studies examining women’s responses to hypoxia.

From a physiological perspective, exogenous estrogenic and progestogenic compounds may influence several systems involved in oxygen uptake, transport, delivery, and utilization [12]. These include ventilatory control, vascular tone, endothelial function, blood pressure regulation, hematological status, and substrate metabolism, all of which are relevant under hypoxic conditions [12, 59, 60]. However, the direction and magnitude of these effects are likely to depend on the contraceptive formulation, the androgenic or anti-androgenic properties of the progestin, the route of administration, duration of use, and whether participants are assessed during active hormone intake or during the hormone-free interval. This distinction is particularly important for combined oral contraceptive users, because the active-pill phase and withdrawal phase do not represent equivalent endocrine states.

Evidence directly examining hormonal contraception in hypoxia remains limited and somewhat inconsistent. In a large study assessing ventilatory response to hypoxia and high-altitude tolerance in women, Richalet et al. [61] reported that oral contraception or hormonal treatment did not significantly affect hypoxic ventilatory response or high-altitude illness scores [61]. This suggests that hormonal contraception may not strongly modify acute hypoxic ventilatory tolerance in all contexts. However, this finding should not be interpreted as evidence that all contraceptive methods are physiologically neutral during hypoxia, because most studies do not systematically differentiate contraceptive type, dose, route of administration, progestin generation, active versus withdrawal phase, or duration of use.

Beyond ventilatory control, hormonal contraception may also influence vascular and hematological determinants of hypoxia tolerance. Oral contraceptive use has been associated with changes in endothelial function and nitric oxide bioavailability, although findings vary according to the vascular bed studied and the hormonal formulation used. These effects could be relevant in hypoxia, where vascular regulation and oxygen delivery become central determinants of physiological tolerance [62]. In addition, hormonal contraceptive use may influence hemoglobin concentration, iron status, and red blood cell parameters, partly through reductions in menstrual blood loss. This may affect oxygen-carrying capacity, although the clinical relevance for hypoxic exposure probably depends on baseline iron status, bleeding profile, contraceptive method, and exposure severity [63].

The methodological implications are important. Grouping all hormonal contraceptive users together may obscure physiologically meaningful differences between combined oral contraceptives, progestin-only pills, injectables, implants, hormonal intrauterine devices, patches, and vaginal rings. Similarly, comparing naturally cycling women with hormonal contraceptive users without documenting menstrual cycle phase, active hormone phase, withdrawal bleeding, formulation, and duration of use can introduce substantial misclassification. Recent methodological recommendations in exercise physiology emphasize the need to distinguish naturally menstruating women from hormonal contraceptive users and to document the specific contraceptive method, because exogenous hormones create endocrine profiles that cannot be assumed to mirror endogenous menstrual cycle phases [64, 65].

Future hypoxia studies should therefore report contraceptive type, formulation, route of administration, duration of use, active or withdrawal phase, bleeding pattern, and, when feasible, circulating estradiol and progesterone concentrations.

### Menopausal status and hypoxia responses

Menopause, marked by the cessation of menstruation and a decline in the production of multiple hormones, including estrogen and progesterone, adds complexities to women’s physiological responses to hypoxia. Estrogen, in particular, plays an important role in maintaining cardiovascular and ventilatory function by promoting vasodilation, improving endothelial function, and facilitating blood flow and oxygen delivery to tissues [29]. The reduction of estrogen in postmenopausal women can therefore lead to diminished ventilatory and cardiovascular adaptations when exposed to a hypoxic environment [66], a finding consistent with observations by Puthon et al. [67] showing that postmenopausal women exhibit a blunted hypoxic ventilatory response during maximal exercise compared with younger women. Nevertheless, recent work indicates that menopause-related hormone decline does not uniformly blunt hypoxia-related physiology across all settings, suggesting that context (e.g., altitude, exposure model) and aging processes may also be decisive modulators [68, 69].

Studies show that postmenopausal women exhibit lower hypoxic ventilatory and heart rate responses compared to premenopausal women and, at high altitude, a higher prevalence of excessive erythrocytosis and elevated chronic mountain sickness scores, suggesting that menopause-related changes in ventilatory compensation and oxygen homeostasis may contribute to greater vulnerability to chronic hypoxia [70]. A study conducted by Moya et al. [66] reported that postmenopausal Tibetan women living at high altitudes demonstrated significantly lower hypoxic ventilatory response (HVR) and hypoxic heart rate response than their premenopausal counterparts, showing that postmenopausal women had a reduced capacity to adapt to hypoxia. By contrast, in controlled normobaric hypoxia, postmenopausal and eumenorrheic women showed no significant differences in HVR, ventilation, heart rate, or acute mountain sickness incidence, underscoring how population, altitude, and methodology can shape observed effects [69].

This decline in estrogen not only affects ventilatory responses but also blunts cardiovascular adaptations necessary for efficient oxygen delivery during hypoxia. Reduced estrogen levels can impair the body’s ability to increase heart rate and promote vasodilation through diminished eNOS activation, reduced NO bioavailability, and altered autonomic regulation, all of which are essential for maintaining oxygen supply to tissues during physical exertion in hypoxic conditions [71]. Accordingly, menopause-related reductions in aerobic capacity reported in normoxic conditions could translate into greater functional limitation when oxygen availability is reduced, although direct comparative data on exercise performance in hypoxia between pre- and postmenopausal women remain limited [72].

Aging and, in females, the loss of estrogen after menopause are associated with reduced induction of cardioprotective heat‑shock proteins [73–75], which may blunt adaptive responses to hypoxic or ischemic stress. Heat shock proteins, particularly HSP72, play an important role in protecting cardiac cells from oxidative stress and damage caused by hypoxia. As women age and transition through menopause, cardioprotective stress responses such as HSP induction may be attenuated, potentially increasing vulnerability to hypoxic stress [76]. Preclinical data support this concept: in an aged ovariectomized rat model, Stice et al. [76] reported a loss of HSP72 induction in response to hypoxia/reoxygenation in cardiomyocytes, and estradiol replacement did not restore this HSP response in aged cells. However, immediate in vivo 17β-estradiol replacement preserved in vivo cardiac contractility and attenuated inflammatory signaling (e.g., TNF-α, IL-1β) in aged ovariectomized rats. These findings provide mechanistic insight, but extrapolation to postmenopausal women exposed to environmental or clinical hypoxia remains limited, given the use of an animal model and severe in vitro hypoxia/reoxygenation conditions. Additionally, estrogen treatment decreases oxidative stress and inflammation in the myocardium via upregulation of antioxidant and cardioprotective pathways, including cystathionine γ-lyase and Per2, highlighting its multifaceted protective effects in estrogen-deficient hypoxic states [76, 77]. Similarly, Laouafa et al. [78] demonstrated that estradiol treatment in animal models exposed to chronic intermittent hypoxia protected against cardiorespiratory dysfunctions and reduced oxidative stress, underscoring estrogen’s protective effects on the cardiovascular and respiratory systems under hypoxic conditions.

While these findings suggest that estrogen plays a key role in mediating hypoxic responses in postmenopausal women, further research is needed to explore the broader impacts of menopause on hypoxia sensitivity. Nishiwaki et al. [71] reported that, within their training protocol, exercise performed under hypoxic conditions improved vascular function and reduced arterial stiffness, whereas no significant change was observed with an intensity-matched normoxic program. However, because the sample included only postmenopausal women, this study does not allow inference about sex- or age/menopause-specific effects. Moreover, aerobic exercise in normoxia is also well established to improve vascular health in this population [79, 80]. Collectively, these findings suggest that hypoxic training may represent an additional stimulus in some contexts rather than the only effective approach for improving vascular function in postmenopausal women. Moreover, menopause’s interaction with hypoxia extends into sleep physiology: observational and mechanistic evidence links menopausal HRT with a lower prevalence of obstructive sleep apnea (OSA), a condition characterized by recurrent intermittent hypoxia, aligning with experimental data showing estrogen’s protection against chronic intermittent hypoxia-induced cardiorespiratory dysfunction [81]. Accordingly, future trials should stratify by age and HRT timing (the “timing hypothesis”, which proposes that the cardiovascular effects of HRT depend critically on the interval between menopause onset and initiation of treatment, with greater benefits when HRT is started early in the postmenopausal period) and test combined HRT–exercise approaches to optimize hypoxia-relevant cardiovascular outcomes [82].

In conclusion, the transition into menopause significantly alters women’s responses to hypoxia through the combined decline in ovarian hormones. While reduced estrogen availability affects vascular and cardiovascular adaptations to hypoxia, the concomitant loss of progesterone may play a central role in the attenuation of ventilatory responses after menopause. Together, these hormonal changes may increase vulnerability to hypoxic environments, particularly at high altitude. Although HRT represents a potential intervention, further studies are required to provide clearer guidelines for women exposed to hypoxic environments or high-altitude activities (Fig. 1). In particular, disentangling the respective contributions of aging, estrogen deficiency, and progesterone loss, and identifying which hypoxic contexts (acute altitude exposure, chronic residence, intermittent sleep-related hypoxia) are most responsive to hormonal and/or exercise-based interventions, should be priority targets for future research.

### Pregnancy as a distinct hormonal and physiological state

Pregnancy represents a distinct physiological condition characterized by profound hormonal changes from early gestation that initiate a cascade of anatomical, physiological, and metabolic adaptations affecting nearly every organ system to support fetal growth while preserving maternal homeostasis [83]. Maternal plasma volume increases by approximately 40–50% over the course of pregnancy, exceeding the concurrent rise in red blood cell mass and resulting in physiological hemodilution despite increased erythropoiesis [84–86]. In parallel, pregnancy is characterized by marked vasodilation, increased uterine and placental blood flow, and elevations in cardiac output and ventilation to meet the heightened oxygen demands of both the mother and the developing fetus [84]. These adaptations are particularly relevant in hypoxic environments, where maternal oxygen uptake, oxygen-carrying capacity, vascular regulation, and uteroplacental perfusion become critical determinants of fetal oxygen delivery.

At high altitude, the reduced barometric pressure and the resulting hypobaric hypoxia impose additional constraints on these pregnancy-related adaptations. Pregnancy at high altitude has been associated with increased risks of adverse maternal and fetal outcomes. Gonzales [87] reported higher rates of congenital malformations, stillbirths, small for gestational age, and preeclampsia among populations living at high altitude, which are associated with elevated maternal hemoglobin levels due to chronic hypoxia. Similarly, Moore et al. [88] found that the incidence of pregnancy-induced hypertension was significantly higher at higher altitudes, increasing from 3.6% at 1,600 m to 15.9% at 3,100 m, with symptoms like proteinuria and edema also more frequent. More recently, Bailey et al. [89] demonstrated that residence at ≥ 2,500 m was associated with a 33% higher combined frequency of hypertensive disorders of pregnancy (HDP) (including gestational hypertension, preeclampsia with or without severe features, eclampsia, and HELLP syndrome) compared with women at lower altitudes. High-altitude residence was also accompanied by higher maternal blood pressures throughout gestation and a greater incidence of related adverse neonatal outcomes — including low birth weight infants (< 2,500 g), lower five-minute Apgar scores (< 7), and increased neonatal intensive care unit (NICU) admissions — with the latter outcomes largely mediated by the increased prevalence of HDP.

The mechanisms underlying these associations appear to involve impaired vascular and placental adaptation to chronic hypoxia. Chronic low-oxygen exposure may alter endothelial homeostasis and vascular regulation during pregnancy, contributing to maladaptive vasodilatory responses, impaired NO signaling, and endothelial dysfunction, which are central features of hypertensive disorders of pregnancy such as preeclampsia [90]. Developmental and chronic hypoxia may also affect fetoplacental endothelial signaling by altering the expression of endothelial genes and disrupting vasodilatory pathways in placental vasculature, thereby compromising uteroplacental blood flow [91]. This failure of maternal and fetoplacental vascular adaptation is central to the pathogenesis of hypertensive disorders of pregnancy, including preeclampsia, and reinforces the mechanistic link between hypoxia, endothelial dysfunction, and adverse pregnancy outcomes [92].

Direct clinical evidence from high-altitude pregnant populations demonstrates impaired endothelial function: flow-mediated dilation (FMD), a standard measure of endothelium-dependent vasodilation, is reduced in women living at very high altitude, supporting the presence of systemic endothelial dysfunction associated with hypoxic exposure [93].

At the placental level, chronic hypoxia and impaired uteroplacental perfusion can disrupt placental vascular function, metabolic regulation, and nutrient transport, thereby contributing to fetal growth restriction and increasing the risk of preeclampsia, even though placental oxygen diffusing capacity itself is often preserved [94–96]. Although pregnancy induces compensatory responses such as increased ventilation, plasma volume expansion, and enhanced erythropoiesis, these adaptations may not fully offset the reduction in oxygen availability at altitude. Elevated maternal hemoglobin levels, potentially indicative of increased red blood cell mass, may not adequately offset reduced oxygen saturation, contributing to adverse pregnancy outcomes by increasing blood viscosity [97].

Evidence from short-term altitude exposure during pregnancy remains more limited than evidence from chronic high-altitude residence. In a survey involving 459 pregnancies, Keyes et al. [98] found that while activities like hiking, running, and swimming were common and often without complications, pregnancies with high-altitude exposure had an increased risk of preterm labor and a higher likelihood of babies needing oxygen at birth. Notably, over half of these pregnancies involved travel to high altitudes, yet most women did not receive medical advice about the associated risks. These findings suggest that brief or recreational altitude exposure during pregnancy should be interpreted separately from chronic high-altitude residence, as the duration of exposure, degree of acclimatization, and underlying maternal adaptation differ substantially between these contexts.

Overall, pregnancy illustrates how a distinct endocrine and physiological condition can modify maternal responses to hypoxic stress through interacting ventilatory, cardiovascular, hematological, endothelial, and placental mechanisms. However, the available evidence should be interpreted according to the exposure model considered: brief or recreational altitude exposure, chronic high-altitude residence, and lifelong or multigenerational high-altitude exposure do not involve the same degree of acclimatization, vascular remodeling, or placental adaptation. Future studies should therefore report altitude level or inspired oxygen fraction, exposure duration, maternal SpO₂, hemoglobin/hematocrit, blood pressure, gestational age, residence or acclimatization history, and placental or fetal outcomes to better clarify how pregnancy-specific adaptations interact with hypoxic stress.

### Androgenic states and gender-affirming hormone therapy: emerging considerations

Despite the well-established influence of hormones on physiological responses to hypoxia, the impact of these mechanisms on hypoxic adaptation in transgender individuals has never been directly studied. Nevertheless, robust findings from Gender-Affirming Hormone Therapy (GAHT) research provide valuable insights into pathways that are central to hypoxia tolerance [99]. GAHT reshapes the hormonal environment and induces shifts in ventilatory, cardiovascular, and erythropoietic responses, three pillars of physiological adaptation to low oxygen.

GAHT induces pronounced hematological remodeling, a key determinant of oxygen transport during hypoxia. Large longitudinal and prospective studies indicate that transgender women (assigned male at birth) receiving feminizing hormone therapy exhibit reductions in hemoglobin and hematocrit, with hematological values shifting toward cisgender female reference ranges, as defined in cohorts on stable hormone therapy [100–102]. Such reductions inevitably lower arterial oxygen-carrying capacity, which may influence tolerance to high-altitude or clinical hypoxia. Conversely, transgender men (assigned female at birth) undergoing testosterone therapy consistently exhibit increased erythropoiesis, with significant rises in hemoglobin and hematocrit and a higher prevalence of testosterone-induced erythrocytosis [103–105]. These changes are expected to enhance oxygen-carrying capacity during hypoxia, although very high hematocrit levels may elevate blood viscosity and impose additional cardiovascular strain, particularly relevant in hypoxic environments where pulmonary pressures are already elevated.

In parallel, GAHT induces cardiovascular changes that may further influence hypoxic responses. Feminizing hormone therapy has been associated with alterations in lipid profiles, endothelial function, blood pressure regulation, and a higher risk of thromboembolic events in some populations [106, 107]. Masculinizing therapy, while increasing lean mass and oxygen transport capacity, may also lead to rises in blood pressure, low-density lipoprotein (LDL), and inflammatory markers [108, 109]. These cardiovascular adaptations could interact with hypoxia, which already increases sympathetic activation, vascular resistance, and cardiac workload, potentially modifying acute and chronic tolerance to low oxygen.

GAHT alters hormonal environments that modulate ventilatory drive. Progesterone and testosterone are known to stimulate ventilation and chemoreceptor sensitivity, whereas estrogen predominantly influences vascular and autonomic regulation. Although no study has yet assessed ventilatory control or HVR after GAHT, these hormonal effects suggest that transgender individuals may exhibit sex-hormone–dependent differences in ventilatory adaptation to low-oxygen environments. The combination of altered hematological profiles and hormone-driven ventilatory modulation, therefore, provides a plausible framework for understanding how GAHT could influence hypoxic responses, even in the absence of direct empirical data.

Altogether, these findings indicate that GAHT may meaningfully shape physiological adaptation to hypoxia through its effects on oxygen transport and potentially respiratory control. However, the magnitude of these effects is likely to vary with GAHT dosage, route of administration, treatment duration, and residual pre-transition physiology. Dedicated studies are urgently needed to determine how GAHT influences ventilatory responses, cardiovascular adjustments, and hematological adaptations during hypoxic stress. Addressing this gap is essential for developing inclusive, evidence-based recommendations for transgender individuals exposed to high altitude or therapeutic hypoxia.

## Integrative synthesis of hormonal status, hypoxic exposure model, and physiological domains

Taken together, the body of available evidence indicates that women’s physiological responses to hypoxia should be interpreted through the interaction between hormonal status, hypoxic exposure model, and the physiological domain considered. Ovarian and exogenous sex steroids may influence several steps of the oxygen cascade, from oxygen uptake and ventilatory control to oxygen transport, vascular delivery, tissue utilization, and reproductive or placental adaptation. Ventilatory control may be particularly sensitive to progesterone-dominant states, such as the luteal phase and pregnancy, through effects on ventilatory drive and chemosensitivity, making this domain especially relevant during acute hypoxia and early acclimatization [14, 30, 38]. Estrogen-related mechanisms, including endothelial nitric oxide signaling, vascular reactivity, blood rheology, and erythroid regulation, may be more apparent in responses involving oxygen delivery, microvascular perfusion, and hematological adaptation, particularly during repeated, chronic, or lifelong hypoxic exposure [28, 49, 50]. Androgenic states, including hyperandrogenism and gender-affirming testosterone therapy, may also influence hypoxia tolerance through effects on erythropoiesis, hemoglobin concentration, hematocrit, iron availability, and blood viscosity [18, 44, 104].

This cross-system perspective also shows that not all physiological domains are equally relevant across hypoxic exposure models. Acute hypoxia primarily challenges chemosensitivity, ventilation, autonomic regulation, heart rate, blood pressure, vascular tone, and arterial oxygen saturation [33, 110]. Repeated or intermittent hypoxia progressively engages ventilatory acclimatization, endothelial regulation, oxidative-stress signaling, metabolic flexibility, and erythropoietic responses [31, 32]. Chronic or lifelong high-altitude exposure additionally involves pulmonary vascular remodeling, excessive erythrocytosis, reproductive function, placental adaptation, fetal growth, and population-level developmental or genetic adaptation [7, 34, 88]. Accordingly, pregnancy, menopause, hormonal contraception, HRT, and gender-affirming hormone therapy should not be interpreted as isolated categories, but as hormonal contexts whose effects depend on the severity, duration, frequency, and physiological setting of hypoxic exposure.

**Fig. 2:**
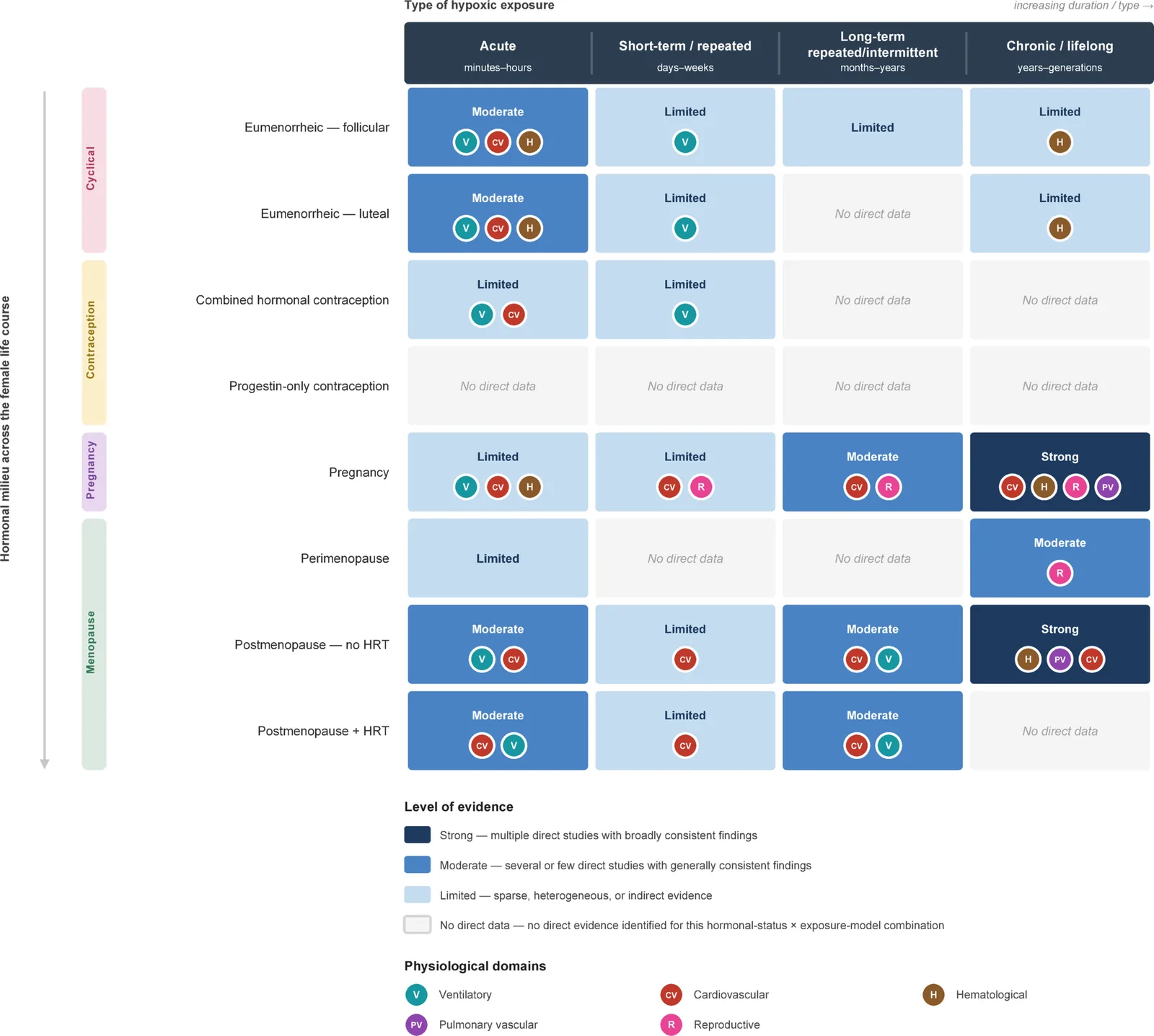
Conceptual framework linking hormonal status, hypoxic exposure model, and physiological response domains in women. Rows represent hormonal or reproductive states across the female life course, while columns represent hypoxic exposure models ranging from acute exposure to chronic or lifelong high-altitude exposure. Cell shading indicates the approximate level of direct evidence available for each hormonal status × exposure model combination. Icons indicate the principal physiological domains documented in the literature: ventilatory, cardiovascular, hematological, pulmonary vascular, and reproductive responses. The figure highlights both areas of relatively stronger evidence, such as pregnancy under chronic or lifelong high-altitude exposure, and major evidence gaps, particularly for progestin-only contraception, HRT, and long-term repeated/intermittent hypoxic exposure. Abbreviations: CV, cardiovascular; H, hematological; HRT, hormone replacement therapy; PV, pulmonary vascular; R, reproductive; V, ventilatory

Overall, the dominant physiological response to hypoxia in women is likely to vary according to both hormonal status and exposure model. Ventilatory responses may be most informative in acute hypoxia and early acclimatization; cardiovascular, autonomic, vascular, and metabolic outcomes appear more context-dependent; hematological and pulmonary vascular responses become increasingly relevant with sustained or chronic hypoxic exposure; and reproductive or placental outcomes should be interpreted primarily within pregnancy-specific, chronic, lifelong, or multigenerational high-altitude contexts. This synthesis supports the need to move beyond broad comparisons between men and women and toward stratified research designs that integrate hormonal status, hypoxic dose, exposure duration, and targeted physiological outcomes. Table 2 summarizes these relationships, while Fig. 2 provides an integrated conceptual framework linking hormonal contexts, hypoxic exposure models, physiological domains, and the approximate level of direct evidence.

**Table 2:** Hormonal states as modulators of women’s physiological responses to hypoxia according to physiological domain, and exposure model

Hormonal state	Main physiological domain(s)	Main reported or hypothesized effect under hypoxia	Key mechanism(s)	Most relevant exposure model
Follicular phase / low-progesterone state	Vascular; ventilatory	Greater estrogen-related vasodilation and tissue oxygen delivery; modest ventilatory stimulation	Increased eNOS/NO bioavailability; lower blood viscosity; reduced progesterone-related ventilatory drive	Acute / repeated
Luteal phase / high-progesterone state	Ventilatory; metabolic	Increased ventilation and potentially enhanced hypoxic ventilatory response	Progesterone-related respiratory stimulation; increased chemosensitivity; possible interaction with HIF signaling	Acute / repeated
Combined hormonal contraception	Ventilatory; vascular; hematological	Generally limited or inconsistent evidence; potential vascular and hematological modulation depending on formulation	Exogenous estrogen/progestin exposure; ovulation suppression; reduced menstrual blood loss	Mostly acute
Progestin-only contraception	Ventilatory; vascular; hematological	Uncertain; likely dependent on progestin type, systemic exposure, and bleeding profile	Variable ovulation suppression; different progestin generation, dose, and route	Mostly acute / insufficient data
Pregnancy	Cardiovascular; ventilatory; hematological; placental/reproductive	Increased ventilation, plasma volume, cardiac output, and uteroplacental oxygen demand; increased vulnerability to fetal growth restriction and hypertensive disorders at altitude	Gestational cardiovascular and ventilatory adaptation; placental vascular regulation; NO-dependent vasodilation; angiogenic balance	Pregnancy-specific hypoxia / chronic high-altitude residence
Menopause / low-estrogen and low-progesterone state	Ventilatory; cardiovascular; vascular; hematological	Potentially blunted HVR and heart-rate response; reduced vascular reactivity; greater vulnerability during chronic altitude exposure	Loss of progesterone-related respiratory drive; reduced eNOS/NO signaling; arterial stiffening; altered erythropoietic regulation	Acute/chronic / lifelong
Hormone replacement therapy	Cardiovascular; vascular; sleep-related intermittent hypoxia	Potentially protective or restorative effects, depending on timing, formulation, and route	Estrogen-related vascular protection; possible modulation of OSA-related intermittent hypoxia; timing hypothesis	Chronic/intermittent sleep-related hypoxia
Gender-affirming hormone therapy — feminizing	Hematological; cardiovascular	Lower hemoglobin/hematocrit toward cisgender female reference ranges; possible reduction in oxygen-carrying capacity	Estrogen/anti-androgen effects on erythropoiesis; reduced hematocrit	No direct hypoxia studies; potentially relevant to acute/chronic hypoxia
Gender-affirming hormone therapy — masculinizing	Hematological; cardiovascular	Increased hemoglobin/hematocrit and possible erythrocytosis; potential increase in oxygen-carrying capacity but also blood viscosity	Testosterone-related stimulation of EPO; reduced hepcidin; increased iron availability	No direct hypoxia studies; potentially relevant to acute/chronic hypoxia

eNOS, endothelial nitric oxide synthase; EPO, erythropoietin; HIF, hypoxia-inducible factor; HRT, hormone replacement therapy; HVR, hypoxic ventilatory response; NO, nitric oxide; OSA, obstructive sleep apnea

## Methodological implications for women’s hypoxia research

As illustrated in Fig. 2, the level of direct evidence varies substantially across hormonal contexts and hypoxic exposure models. This heterogeneity underscores the need for more standardized methodological approaches in women’s hypoxia research, particularly regarding hormonal characterization, hypoxic dose reporting, exposure context, and outcome selection. A major limitation of the current literature is that female participants are often included without sufficient information on menstrual cycle phase, contraceptive use, menopausal status, hormone therapy, pregnancy status, or gender-affirming hormone therapy. This lack of stratification may contribute to inconsistent findings across studies, particularly when acute hypoxic responses, repeated exposure, chronic altitude residence, and lifelong high-altitude adaptation are interpreted within the same framework. Future research should therefore systematically report the hormonal context of participants and the characteristics of the hypoxic stimulus, including altitude level or inspired oxygen fraction, oxygen saturation, exposure duration, exposure frequency, cumulative hypoxic dose, and whether exposure occurs at rest, during sleep, or during exercise.

For naturally cycling women, menstrual cycle verification should be adapted to the aims and constraints of the study. Calendar-based classification is feasible and inexpensive but remains vulnerable to cycle variability and phase misclassification, particularly when used without biological confirmation. More robust approaches include standardized menstrual bleeding logs, ovulation testing through urinary luteinizing hormone detection, and basal body temperature monitoring to support luteal-phase identification. When the research question specifically concerns estrogen- and progesterone-dependent mechanisms, hormonal assays should be prioritized, including serum or salivary estradiol and progesterone measurements [111]. These approaches allow a more accurate interpretation of cycle-related effects and make it possible to move beyond broad phase-based categories toward consideration of the estrogen/progesterone ratio. When ovulation timing is central to the protocol, ultrasound confirmation may provide the highest level of validity, although this approach increases participant burden, cost, and logistical complexity.

Hormonal contraception should also be reported in detail rather than treated as a single category. Studies should distinguish combined hormonal contraceptives from progestin-only methods and should specify the delivery system, including oral pills, transdermal patches, vaginal rings, implants, injectables, and hormonal intrauterine devices. The formulation, estrogen dose, progestin type, androgenic or anti-androgenic properties, duration of use, and timing of assessment within the active or hormone-free interval should be reported when applicable. This level of detail is essential because different contraceptive methods may have distinct effects on ventilatory control, vascular regulation, blood pressure, substrate metabolism, and hematological responses under hypoxic conditions.

In postmenopausal women, menopausal status should be carefully characterized rather than inferred from age alone. Studies should report age, time since menopause, criteria used to define menopausal status, and relevant cardiometabolic characteristics. HRT requires specific consideration, including type of therapy, estrogen and/or progestogen formulation, route of administration, dose, duration of use, and timing of initiation relative to menopause. This distinction is important because the physiological effects of HRT may vary according to the timing of initiation and may influence vascular, ventilatory, hematological, and metabolic responses to hypoxia [112]. Similarly, pregnancy status should be explicitly reported, and pregnant participants should not be grouped with non-pregnant women because pregnancy represents a distinct endocrine, respiratory, cardiovascular, hematological, and metabolic state.

In parallel with hormonal characterization, hypoxic exposure should be described with sufficient precision to allow interpretation and comparison across studies. At minimum, studies should report the exposure model, altitude level or inspired oxygen fraction, normobaric or hypobaric condition, exposure duration, exposure frequency, number of sessions, oxygen saturation response, and cumulative hypoxic dose. When relevant, arterial oxygen saturation, peripheral oxygen saturation, or arterial blood gases should be reported to characterize the individual physiological dose (internal hypoxic dose) rather than relying only on altitude or inspired oxygen fraction (external hypoxic dose) [113]. The context of exposure should also be specified, because hypoxia at rest, during exercise, or during sleep engages different physiological systems and may interact differently with hormonal status.

Finally, future studies should clearly indicate the level of evidence supporting hormonal and hypoxic exposure classification. For menstrual cycle research, this may range from calendar-based estimation to biological confirmation through luteinizing hormone testing and hormonal assays. For hypoxic exposure, this may range from altitude or inspired oxygen fraction alone to individualized oxygen saturation profiles and cumulative dose metrics. Transparent reporting of these methodological details would improve reproducibility, facilitate comparison across studies, and allow more precise evaluation of how hormonal status modulates ventilatory, cardiovascular, vascular, hematological, muscular/metabolic, and reproductive responses to hypoxia. Such methodological refinement is essential for developing evidence-based recommendations for women exposed to hypoxic environments in clinical, occupational, athletic, and high-altitude contexts.

## Conclusion

This review highlights that women’s physiological responses to hypoxia cannot be interpreted independently of hormonal status, reproductive life-course stage, and hypoxic exposure model. Menstrual cycle phase, hormonal contraception, pregnancy, menopause, HRT, and gender-affirming hormone therapy may each influence components of the oxygen cascade, including ventilatory control, cardiovascular and autonomic regulation, vascular function, hematological responses, blood rheology, muscular/metabolic regulation, and reproductive or placental outcomes. However, these effects are unlikely to be uniform across all hypoxic contexts. Acute hypoxia primarily challenges ventilatory, autonomic, cardiovascular, and oxygenation responses, whereas repeated, chronic, lifelong, or multigenerational exposure progressively involves acclimatization, plasma volume regulation, erythropoiesis, vascular remodeling, placental adaptation, and developmental or population-level influences.

The body of available evidence remains limited and methodologically heterogeneous, particularly for hormonal contraception, HRT, gender-affirming hormone therapy, muscular/metabolic responses, and long-term repeated or intermittent hypoxic exposure. Sleep-related intermittent hypoxia also deserves greater attention, particularly in postmenopausal women and HRT users, because nocturnal oxygenation may represent an additional context in which hormonal status modifies hypoxic vulnerability. Future studies should therefore move beyond broad comparisons between men and women and systematically characterize hormonal status, including menstrual cycle phase verification, contraceptive formulation, menopausal status, hormone therapy timing, pregnancy status, and exogenous hormone exposure. In parallel, external and internal hypoxic dose should be reported with precision, including altitude level or inspired oxygen fraction, oxygen saturation response, exposure duration, exposure frequency, cumulative dose, and whether exposure occurs at rest, during sleep, or during exercise. Such methodological refinement is essential to clarify women-specific hypoxic responses and to support evidence-based recommendations for clinical, occupational, athletic, and high-altitude settings.

## Data Availability

No datasets were generated or analysed during the current study.

## Abbreviations

ALAS2: 5-aminolevulinate synthase 2
APGAR: Appearance, Pulse, Grimace, Activity, Respiration (newborn score)
BCL-xL: B-cell lymphoma-extra large (anti-apoptotic protein)
CD235a: Cluster of Differentiation 235a (glycophorin A; erythroid marker)
eNOS: Endothelial nitric oxide synthase
EPO: Erythropoietin
EPOR: Erythropoietin receptor
ERα / ERβ: Estrogen receptor alpha/beta
FSH: Follicle-stimulating hormone
FMD: Flow-mediated dilation
GAHT: Gender-affirming hormone therapy
GnRH: Gonadotropin-releasing hormone
GATA-1: GATA-binding factor 1 (erythroid transcription factor)
HDP: Hypertensive disorders of pregnancy
HELLP: Hemolysis, Elevated Liver enzymes, Low Platelets (syndrome)
HIF-1α / HIF-2α: Hypoxia-inducible factor 1 alpha / 2 alpha
HPG axis: Hypothalamic–pituitary–gonadal axis
HSC: Hematopoietic stem cell
HSP72: Heat shock protein 72
HRT: Hormone replacement therapy
HVR: Hypoxic ventilatory response
IL-1β: Interleukin-1 beta
LDL: Low-density lipoprotein
LH: Luteinizing hormone
mRNA: Messenger RNA
NICU: Neonatal intensive care unit
NO: Nitric oxide
O₂: Molecular oxygen
PCOS: Polycystic ovary syndrome
Per2: Period circadian regulator 2 (clock gene/protein)
TNF-α: Tumor necrosis factor alpha
USA: United States of America
